# Analysis of patients scheduled for neoadjuvant therapy followed by surgery for esophageal cancer, who never made it to esophagectomy

**DOI:** 10.1186/s12957-019-1630-8

**Published:** 2019-05-27

**Authors:** Lieven Depypere, Melissa Thomas, Johnny Moons, Willy Coosemans, Toni Lerut, Hans Prenen, Karin Haustermans, Hans Van Veer, Philippe Nafteux

**Affiliations:** 1 Department of Thoracic Surgery, University Hospitals Leuven, Herestraat 49, 3000 Leuven, Belgium; 2 Department of Radiation Oncology, University Hospitals Leuven, Leuven, Belgium; 3 Department of Oncology, University Hospital Antwerp, Edegem, Belgium; 4 Department of Chronic Diseases, Metabolism and Ageing, KU Leuven, Leuven, Belgium; 5 Department of Oncology, KU Leuven, Leuven, Belgium

**Keywords:** Esophageal neoplasms, Neoadjuvant therapy, Treatment outcome, Adverse effects

## Abstract

**Background:**

Neoadjuvant treatment followed by esophagectomy is standard practice in locally advanced esophageal cancer. However, not all patients who started neoadjuvant treatment will undergo esophageal resection. The purpose of our study was to investigate the group of patients, scheduled for neoadjuvant treatment followed by esophagectomy, who never made it to esophageal resection.

**Methods:**

We retrospectively analyzed patients treated between 2002 and 2015 for locally advanced esophageal cancer, who did not undergo esophagectomy after neoadjuvant treatment. Subanalysis was performed according to time period (2002–2010 versus 2011–2015) and histology (adenocarcinoma versus squamous cell carcinoma).

**Results:**

In 114 of 679 patients (16.8%), surgery was not performed after neoadjuvant treatment. Reasons for cancelation were disease progression (50 patients, 43.9%), poor general condition (26 patients, 22.8%), irresectability (14 patients, 12.3%), patients’ own decision (15 patients, 13.2%), and death during neoadjuvant treatment (9 patients, 7.9%). In the second time period, there were less irresectable tumors (17.7% versus 5.8%; *p* = 0.044). Median overall survival was not different over time (9.2 versus 12.5 months; *p* = 0.937). Irresectability (*p* = 0.032), patients’ refusal (*p* = 0.012), and poor general condition (*p* = 0.002) were more frequent as reasons for cancelation in squamous cell carcinoma patients. Median overall survival was, respectively, 12.5 and 9.9 months for adenocarcinoma and squamous cell carcinoma patients (*p* = 0.441). The majority of patients refusing surgery had a clinical complete response (73.3%). They had a median overall survival of 33.2 months.

**Conclusions:**

One in six patients starting neoadjuvant treatment for locally advanced esophageal cancer never made it to esophagectomy, more than half of them for oncological reasons, but also 1.3% because of death during treatment. Over time, irresectability as reason decreased. As a result, the relative weight of medical inoperability increased, indicating the importance of upfront testing of medical operability. Cancelation of surgery was significantly more common in patients with a squamous cell carcinoma, and this histology seems to represent a more complex oncological and functional entity. Refusal of esophagectomy based on clinical complete response showed a significant survival benefit compared to those who did not undergo esophagectomy because of other reasons.

## Background

Primary surgery has long been the mainstay of treatment in patients with locally advanced esophageal cancer (EC). In a quest to improve outcome, several neoadjuvant strategies using chemotherapy and chemoradiotherapy have been investigated [1]. Since randomized controlled trials showed a better local tumor control and a survival benefit in patients undergoing neoadjuvant treatment (NT) [2–9], a multimodality approach is now considered standard of care in the treatment of locally advanced EC [10, 11]. However, up to 20% of the patients who start NT will not undergo surgery for reasons such as disease progression, medical inoperability, and patients’ own decision [12, 13]. Furthermore, mortality among patients during NT is around 1 to 3% [2–9, 12].

The aim of this study was to evaluate the group of patients, scheduled for NT followed by surgery, who never made it to esophageal resection (ER), in a clinical series apart from any trial. The following clinical questions were considered: what is the mortality of NT, how many patients will not be operated after NT, what are the reasons for cancelation of surgery, and how can we reduce this number?

## Methods

Approval from the Ethical Committee at the University Hospital Leuven was obtained for this study (S59162). Between 2002 and 2015, 679 consecutive patients with locally advanced resectable EC started NT either in our institution or at a referring hospital, due to receive subsequently ER in our department. All patients were discussed in our EC multidisciplinary tumor board (MTB) meeting.

All patients underwent an endoscopy with biopsy for histological proof of malignancy. Clinical staging included endoscopic ultrasound (EUS) when possible (patency of the tumor), a computed tomography (CT) scan of the chest and abdomen, and a fluorodeoxyglucose positron emission tomography with integrated computed tomography (FDG-PET/CT). When needed, other examinations such as bronchoscopy, endobronchial ultrasound (EBUS), ultrasonography, magnetic resonance imaging (MRI), bone scintigraphy, mediastinoscopy, thoracoscopy, and/or laparoscopy were performed. Patients were clinically staged according to the American Joint Committee on Cancer TNM staging system, seventh edition [14].

Neoadjuvant regimen (chemotherapy versus chemoradiation therapy (up to 45 Gy) and type of chemotherapy) was discussed during a MTB meeting in our hospital in the referring center, depending on TNM classification, tumor histology, location of the tumor, and an estimation of the patient’s functional status. Therefore, indications can differ between centers.

Patients with suspected lymph nodes below the celiac trunk and cT4 tumors with invasion in the aorta of more than 25% of the circumference or visible ingrowth in the trachea during bronchoscopy were excluded. Also, patients undergoing definitive chemoradiation therapy (> 50 Gy) were excluded.

Restaging after NT was performed with a CT scan of the chest and abdomen or a FDG-PET/CT. Preoperative functional assessment always included pulmonary function tests and a cardiac stress test. Other investigations were on indication.

Of all patients starting with NT but not proceeding to surgery (NT−S group), both tumor (histology, cTNM at time of diagnosis, and tumor location), patient (age, gender), and treatment-related (NT regimen) characteristics were collected. Furthermore, the reason for canceling ER and the clinical response to NT were retrospectively reviewed in this patient cohort. Clinical complete response (cCR) was defined as a standardized uptake value (SUV) of zero or an inflammatory pattern on FDG-PET/CT combined with a negative endoscopic examination with biopsy. Clinical partial response (cPR) was defined as a reduced tumor volume on CT or reduced SUV on PET without any signs of progression in other locations. Stable disease was defined as no visible change in volume on CT and almost the same SUV on PET. Irresectability was defined as the impossibility to perform a macroscopic complete resection at time of reevaluation after NT or at the time of surgery because of ingrowth in adjacent structures that could not be removed.

All patients (those who underwent esophageal resection and also patients who did not) were offered best medical treatment (systemic therapy, radiotherapy, or surgery) in case of recurrent disease, as discussed in the MTB meeting.

Since the indications for NT in our institution and referring hospitals changed over the time period of investigation, the NT−S patient cohort was divided in two groups: from 2002 until 2010, where NT was mainly reserved for bulky lymph node involvement, or cT4 tumors and from 2011 until 2015, in which NT was the standard therapy for all tumors with a clinical staging above cT2N0. Patients were also stratified according to histology: squamous cell carcinoma (SCC) versus adenocarcinoma (AC).

Median overall survival (OS) was calculated (from the date of histological proof of the esophageal carcinoma) for the entire patient cohort and according to the two time frames and histology.

## Statistics

Summary statistics were presented as means and range or medians and interquartile range (IQR) for continuous variables and as frequencies and percentages for categorical variables. These variables were compared between groups with unpaired *t* tests.

Patient survival was estimated by Kaplan-Meier curves from the date of histological diagnosis and compared by log-rank tests. A *p* value smaller than 0.05 was considered as significant.

The analyses were repeated for the patients in two time frames and according to tumor histology.

All analyses were performed using IBM SPSS Statistics software, version 24 (SPSS Inc., Chicago, IL, USA).

## Results

Patient, tumor, and treatment-related characteristics of all patients are described in Table 1. Of 679 patients with locally advanced resectable EC scheduled for NT followed by surgery, 114 patients (16.8%) did not undergo an ER (NT−S group). Patients were younger in the group undergoing ER (NT+S group; median 62 years (IQR 55–69) versus 67 years (IQR 60–74), respectively; *p* = 0.002) and had a significantly higher incidence of adenocarcinoma (AC) (66.9% versus 45.6%, respectively; *p* < 0.001). Clinical stage (cT and cN) and type of NT were not significantly different between the two groups (respectively, *p* = 0,400, *p* = 0.862, and *p* = 0.140). In the NT−S group, a cCR was obtained in 23 of 114 patients (20.2%) and 33 patients (28.9%) had a cPR. Nine patients (7.9%) had clinical stable disease and 39 patients (34.2%) a clinical progressive disease. In 10 of the 114 patients (8.8%), clinical restaging was not executed or not interpretable.

**Table 1:** Tumor, patient, and treatment-related characteristics. *NT+S* neoadjuvant treatment plus surgery, *NT−S* neoadjuvant treatment without surgery, *IQR* interquartile range, *AC* adenocarcinoma, *SCC* squamous cell carcinoma, *nCRT* neoadjuvant chemoradiotherapy, *nCT* neoadjuvant chemotherapy

	NT+S		NT−S		All	*p*
No. of patients	565	83.2%	114	16.8%	679	
Gender						
Male	433	76.6%	89	78.1%	522	0.808
Female	132	23.4%	25	21.9%	157	
Age						
Mean (range)	61 (27–83)		65 (32–86)		62 (27–86)	0.002
Time frame						
Before 2011	274	48.5%	62	54.4%	336	0.148
From 2011	291	51.5%	52	45.6%	343	
Histology						
AC	378	66.9%	52	45.6%	430	< 0.0001
SCC	187	33.1%	62	54.4%	249	
Clinical stage						
cT1	2	0.4%		0.0%	2	0.400
cT2	55	9.8%	7	6.1%	62	
cT3	433	77.1%	87	76.3%	520	
cT3–4	38	6.8%	14	12.3%	50	
cT4	33	5.9%	6	5.3%	45	
Clinical N						
cN negative	53	9.4%	11	9.6%	64	0.862
cN positive	512	90.6%	103	90.4%	615	
Type of treatment						
nCRT	491	86.9%	93	81.6%	584	0.140
nCT	74	13.1%	21	18.4%	95	

Reasons for canceled surgery were disease progression in 50 patients (43.9%, 7.4% of all patients), poor general condition (i.e., medical inoperable) in 26 patients (22.8%, 3.8 of all patients), irresectability at time of reevaluation after NT or at the time of surgery in 14 patients (12.3%, 2.1% of all patients ), patients’ own decision in 15 patients (13.2%, 2.2% of all patients), and death during NT in nine patients (7.9%, 1.3% of all patients) (Table 2). The main reason for disease progression was the occurrence of interval metastases in 46 of 50 patients (92.0%) and in four patients (8.0%) progression of distant lymph nodes. Reasons for irresectability were two patients with a tracheo-esophageal fistula, four with pancreas invasion, one with aortic arch invasion, three with tracheal invasion, one with invasion of the left mainstem, and three with invasion of the descending aorta. Of the 15 patients refusing esophagectomy, one had a simultaneous lung tumor, three had a cPR, and 11 of 15 patients (73.3%) had a clinical cCR after NT. Of the nine deceased patients, causes of death were sepsis in five patients, myocardial infarction in two patients, subarachnoidal bleeding in one patient, and trachea-esophageal fistula in one patient.

**Table 2:** Reasons for cancelation of esophagectomy according to time period and histology. *AC* adenocarcinoma, *SCC* squamous cell carcinoma

Reasons	2002–2010		2011–2015		Total (%)
	AC	SCC	AC	SCC	
Death during treatment	0	4	4	1	9 (7.9)
Disease progression	14	13	15	8	50 (43.9)
Irresectability	3	8	2	1	14 (12.3)
Own decision	4	5	1	5	15 (13.2)
Poor general condition	3	8	6	9	26 (22.8)
Total	24	38	28	24	114
Reasons	AC	SCC	2002–2010	2011–2015	
Death during treatment	4	5	4	5	
Disease progression	29	21	27	23	
Irresectability	5^a^	9^a^	11^b^	3^b^	
Own decision	5^c^	10^c^	9	6	
Poor general condition	9^d^	17^d^	11	15	
Total	52	62	62	52	

^a^*p* = 0.032

^b^*p* = 0.044

^c^*p* = 0.018

^d^*p* = 0.002

**Fig. 1:**
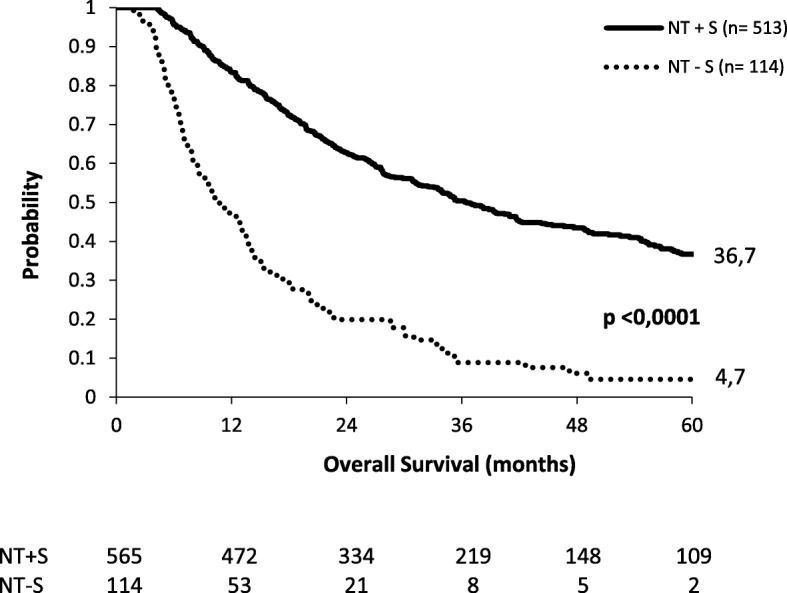
Overall survival in the NT+S and NT−S cohorts. NT+S neoadjuvant treatment plus surgery, NT−S neoadjuvant treatment without surgery

**Fig. 2:**
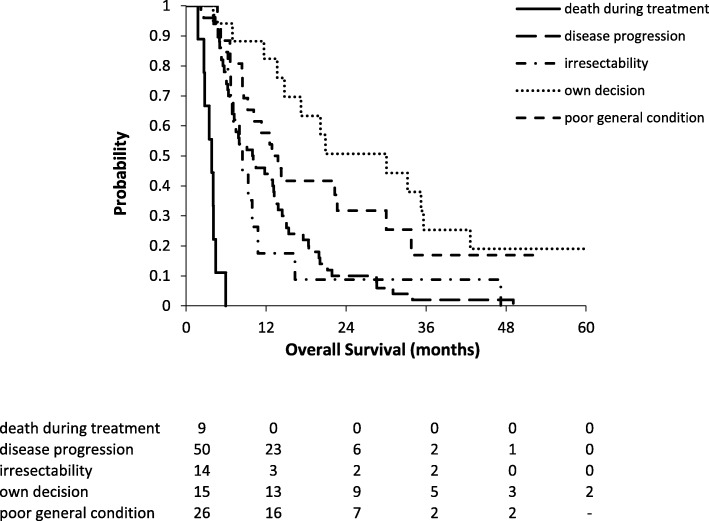
Overall survival in the cohort of patients with canceled esophagectomy

Median OS of patients in the NT+S group and NT−S group was, respectively, 36.5 months and 10.5 months. Five-year OS was, respectively, 36.7% and 4.7% (Fig. 1). In the NT−S cohort, patients who refused esophagectomy had a significant survival benefit (*p* < 0.0001) with a median OS of 33.2 months compared to 12.9 months for those who did not undergo esophagectomy because of poor general condition, 9.9 months for those whose disease progressed, 8.5 months for those who were irresectable, and 3.8 months for those who died during NT (Fig. 2).

**Fig. 3:**
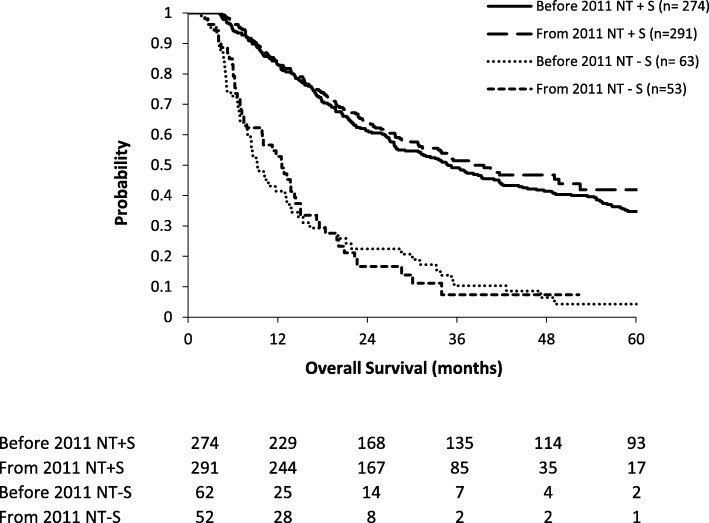
Overall survival in the NT+S and NT−S cohorts, divided into the 2 time frames (2002–2010 and 2011–2015). NT+S neoadjuvant treatment plus surgery, NT−S neoadjuvant treatment without surgery

The NT−S cohort was divided in two time frames (2002–2010 and 2011–2015) (Table 3). There were no significant differences in tumor, patient, and treatment-related characteristics between the two time frames, besides 6 patients with a cT4 tumor between 2002 and 2010 and none between 2011 and 2015. Respectively, 62 of 336 patients in the first time period (18.5%) and 52 of 343 patients in the second time period (15.2%) did not undergo an ER. In the second time period, there were significantly less irresectable tumors (17.7% versus 5.8%, *p* = 0.044). Other reasons for canceled esophagectomy remained the same. Median OS of NT−S patients was not different over time (*p* = 0.937) with 9.2 and 12.5 months, respectively (Fig. 3). Median OS of NT+S patients was 34.8 months in the first time period and 38.3 months in the second time period (*p* = 0.323).

**Table 3:** Tumor, patient, and treatment-related characteristics of patients undergoing neoadjuvant treatment without surgery per time cohort. *IQR* interquartile range, *AC* adenocarcinoma, *SCC* squamous cell carcinoma, *nCRT* neoadjuvant chemoradiotherapy, *nCT* neoadjuvant chemotherapy

		Before 2011	From 2011	All	
Patients	*n* (%)	62 (54.4)	52 (45.6)	114	
Gender					*p* = 0.342
Male	*n* (%)	47 (75.8)	42 (80.8)	89	
Female	*n* (%)	15 (24.2)	10 (19.2)	25	
Age	Median (IQR) Mean (range)	65 (58–74) 64 (35–84)	68 (61–74) 67 (32–86)	67 (60–74) 65 (32–86)	*p* = 0.224
Histology					*p* = 0.077
AC	*n* (%)	24 (38.7)	28 (53.8)	52	
SCC	*n* (%)	38 (61.3)	24 (46.2)	62	
Clinical stage					*p* = 0.131
cT2	*n* (%)	4 (6.5)	3 (5.8)	7	
cT3	*n* (%)	45 (72.6)	42 (80.8)	87	
cT3–4	*n* (%)	7 (11.3)	7 (13.5)	14	
cT4	*n* (%)	6 (9.7)	0 (0.0)	6	
cN status					*p* = 0.173
cN negative	*n* (%)	4 (6.5)	7 (13.5)	11	
cN positive	*n* (%)	58 (93.5)	45 (86.5)	103	
Treatment type					*p* = 0.513
nCRT	*n* (%)	51 (82.3)	42 (80.8)	93	
nCT	*n* (%)	11 (17.7)	10 (19.2)	21

**Fig. 4:**
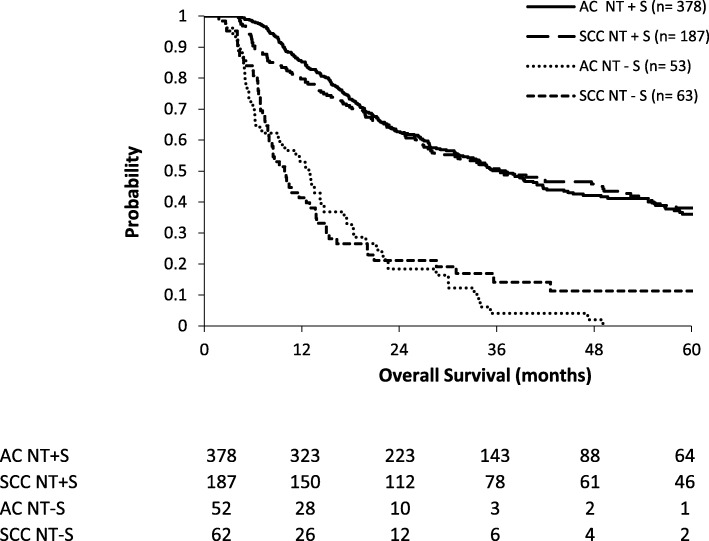
Overall survival in the NT+S and NT−S cohorts, divided per histology (AC and SCC). NT+S neoadjuvant treatment plus surgery, NT−S neoadjuvant treatment without surgery, AC adenocarcinoma, SCC squamous cell carcinoma

The NT−S group was also stratified according to histology (Table 4). Clinical staging and type of NT were significantly different between SCC and AC (*p* = 0.009 and *p* = 0.008, respectively). Respectively, 52 of 430 AC patients (12.1%) and 62 of 249 SCC patients (24.9%) did not undergo an ER. Irresectability (*p* = 0.032), patients’ refusal (*p* = 0.0117), and poor general condition (*p* = 0.002) were significantly more frequent as reasons for canceled esophagectomy in SCC patients compared to AC patients. Median OS was 12.5 months for AC and 9.9 months for SCC (*p* = 0.441) (Fig. 4). Median OS of NT+S patients was not significantly different between AC and SCC, respectively, 37.1 and 36.5 months (*p* = 0.779).

**Table 4:** Tumor, patient, and treatment-related characteristics of patients undergoing neoadjuvant treatment without surgery per histology. *AC* adenocarcinoma, *SCC* squamous cell carcinoma, *IQR* interquartile range, *nCRT* neoadjuvant chemoradiotherapy, *nCT* neoadjuvant chemotherapy

		AC	SCC	ALL	
Patients	*n* (%)	52 (45.6)	62 (54.4)	114	
Gender					*p* = 0.093
Male	*n* (%)	44 (84.6)	45 (72.6)	89	
Female	*n* (%)	8 (15.4)	17 (27.4)	25	
Age	Median (IQR) Mean (range)	67 (62–74) 67 (35–86)	64 (59–72) 64 (32–84)	67 (60–74) 65 (32–86)	*p* = 0.178
Time frame					*p* = 0.077
Before 2011	*n* (%)	24 (46.2)	38 (61.3)	62	
From 2011	*n* (%)	28 (53.8)	24 (38.7)	52	
Clinical stage					*p* = 0.009
cT1–2	*n* (%)	2 (3.8)	5 (8.1)	7	
cT3	*n* (%)	47 (90.4)	40 (64.5)	87	
cT3–4	*n* (%)	2 (3.8)	12 (19.4)	14	
cT4	*n* (%)	1 (1.9)	5 (8.1)	6	
cN status					*p* = 0.752
cN positive	*n* (%)	48 (92.3)	55 (88.7)	103	
cN negative	*n* (%)	4 (7.7)	7 (11.3)	11	
Treatment type					*p* = 0.008
nCRT	*n* (%)	37 (71.2)	56 (90.3)	93	
nCT	*n* (%)	15 (28.8)	6 (9.7)	21

## Discussion

NT followed by ER is considered standard of care in the treatment of locally advanced EC, based on the results of large randomized controlled trials [2–9]. However, up to 22% of the patients who start NT will not undergo surgery [12, 13]. This is in line with our clinical, daily practice series, where 16.8% of the patients scheduled for NT never underwent ER. The purpose of the current study was to evaluate the characteristics of this patient cohort, as well as according to two time frames (2002–2010 and 2011–2015) and according to histology (SCC and AC). We analyzed the failure and mortality rate of NT, the reasons for cancelation of esophagectomy, and how the number of patients not going to surgery can be reduced.

The main reason for canceled esophagectomy was oncological, i.e., disease progression or irresectability. Over time, in our cohort, patients who were irresectable after NT significantly reduced from 3.3% to only 0.9%. This could possibly be related to better staging procedures, more efficient NT in locally advanced resectable EC, or more uniform (contra-)indications for NT. However, this observation might also be due to a histological bias, with—although not significant—less SCC in the second time cohort. It is worth mentioning that five of the six patients with a cT4 in the first time cohort had a SCC despite exclusion of patients with invasion in the aorta or trachea. A reason therefore might be a shift towards definitive chemoradiation therapy in those patients during the second time period. Overall, the number of futile surgical procedures seems to decrease to almost nil which is a major step forward compared to up to 13% irresectable tumors in primary surgery series [2–8]. Disease progression was seen in 7.4% of patients after NT and was not different between the two time cohorts or according to histology. This is similar to the data reported in the literature, i.e., 5–7% [2–8, 15–17]. Whether disease progression is a surrogate for true treatment failure remains an open question. Based on the assumption that undetectable metastatic disease was already present at the time of diagnosis, oncological reevaluation after NT can be interpreted as a natural selection of patients in whom unsuccessful surgery can be omitted [15].

Besides oncological reasons, there are three other causes for canceling surgery. Nine patients (1.3%) died during NT, and this was balanced between the two time cohorts. This is in line with mortality rates reported in the literature [2–9], but it should also be compared with the surgical mortality rates: the 30-day mortality after esophagectomy in our institution is 1.5%, so adding neoadjuvant therapy could be seen as doubling the mortality risk. Secondly, by decreasing irresectability as reason for cancelation of surgery, the relative weight of medical reasons becomes more important, such as poor medical condition, making treatment-related toxicity, and potentially worsening of existing comorbidities’ potential culprits. Negative effects of NT on the heart and lungs are well known [18–23]. Moreover, in the era of “standard” NT, more and more patients start NT without objective documentation of medical fitness and thereby missing upfront medical inoperable patients [24]. Indeed, in this series, 45% of patients undergoing NT, who did not undergo ER, did not have a pretreatment lung function test and only 15% underwent cardiopulmonary exercise testing. Finally, 15 patients (2.2%) refused surgery after NT, which is also in line with results of prospective series [2–9]. The main reason was the achievement of a cCR. Obviously, a cCR is expected to show a significant survival benefit compared to those who did not undergo esophagectomy for other reasons.

Cancelation of ER after NT was significantly more common in patients with SCC, especially for non-oncological reasons such as medical inoperability and patients’ own decision, besides a higher number of irresectable tumors. This emphasizes the difference between SCC versus AC. Therefore, they should probably be considered as a more complex oncological entity especially when dealing with advanced cT3–4 tumors anatomically located between the aorta and pars membranacea which translates into a higher number of patients who are irresectable, and more complex functional entity often due to the use of tobacco and alcohol, translating into medical unfitness. The other reason of higher rate of cancelation of ER in SCC is the refusal based on cCR refusal to undergo ER (two in five patients with an AC and nine in 10 patients with a SCC).

Median OS did not differ between the two time frames and according to histology. Patients in the NT+S cohort had a longer OS in contrast to the NT−S group, given the main reasons for cancelation were disease progression and poor general condition.

Limitations inherent to retrospective analyses also apply to this study. Although a multidisciplinary decision to cancel the surgical treatment was made, the underlying reason was not always mentioned. Furthermore, the number of patients in the NT−S group might be an underestimation as we only included patients who were discussed in our MTB, treated in or referred to our center. Patients initially discussed at an MTB outside our hospital who started NT, with the intention for a referral to our center but who were no longer candidates for surgical treatment, were not included in this study. Also, not included in this study were the patients in whom definitive chemoradiation therapy was given after MTB decision. This therapeutic option could also have affected the numbers and outcomes in NT−S and NT+S groups, especially in SCC patients.

This study raised an important question: “what should I tell my patient before start of NT?” Surgeons, radiation oncologists, and oncologists must collaborate to provide patients correct information after discussing the optimal treatment [12]. Adequate staging before and after NT, including a functional assessment at these two time points, is essential for decreasing the risk of cancelation of ER. Furthermore, information about the risk of disease progression, jeopardizing ER, should be mentioned, since this alters the prognosis of the patient. The potential toxicity of NT should be discussed upfront, especially as patients focus on their quality of life, both during and after treatment [25]. All decisions in the progress of the treatment have to be supported by an MTB meeting which is proven to optimize therapeutic decision-making and outcomes [26]. We hope that the concentration of care in centers for complex esophageal surgery, as initiated by our federal government, will lead to better results in the future [27].

## Conclusions

One in six patients scheduled for NT followed by ER for locally advanced EC never made it to ER. Reasons for canceled surgery were disease progression; medical inoperability; irresectability; patients’ own decision, in particular in the event of a cCR; and mortality during NT. Over time, irresectability as reason decreased, possibly related to a more performant staging and more uniform (contra-)indications for NT over time. As a result, contraindications for surgery based on new or worsening of existing comorbidities become more important. Cancelation of ER after NT was significantly more common in patients with SCC, especially for non-oncological reasons such as medical inoperability and patients’ own decision, besides a higher number of irresectable tumors. Those patients who refused esophagectomy, because of cCR, had a significant survival benefit compared to those who did not undergo esophagectomy because of other reasons.

In general, adequate pretreatment oncological staging and functional assessment together with accurate patient information are pivotal in curative treatment of EC.

## Data Availability

The datasets used and/or analyzed during the current study are available from the corresponding author on reasonable request.

## Abbreviations

AC: Adenocarcinoma
cCR: Clinical complete response
cPR: Clinical partial response
CT: Computed tomography
EBUS: Endoscopic bronchial ultrasound
EC: Esophageal cancer
ER: Esophageal resection
EUS: Endoscopic (esophageal) ultrasound
FDG-PET/CT: Fluorodeoxyglucose positron emission tomography with integrated computer tomography
IQR: Interquartile range
MRI: Magnetic resonance imaging
MTB: Multidisciplinary tumor board
NT: Neoadjuvant therapy
NT+S: Neoadjuvant therapy followed by surgery
NT−S: Neoadjuvant therapy not followed by surgery
OS: Overall survival
SCC: Squamous cell carcinoma
SUV: Standardized uptake value

## References

[1] Sjoquist KM, Burmeister BH, Smithers BM (2011). Survival after neoadjuvant chemotherapy or chemoradiotherapy for resectable oesophageal carcinoma: an updated meta-analysis. Lancet Oncol.

[2] Kelsen DP, Ginsberg R, Pajak TF (1998). Chemotherapy followed by surgery compared with surgery alone for localized esophageal cancer. N Engl J Med.

[3] Kelsen DP, Winter KA, Gunderson LL (2007). Long-term results of RTOG trial 8911 (USA Intergroup 113): a random assignment trial comparison of chemotherapy followed by surgery compared with surgery alone for esophageal cancer. J Clin Oncol.

[4] Surgical resection with or without preoperative chemotherapy in oesophageal cancer: a randomised controlled trial. Lancet. 2002;359:1727–33. 2002/06/07. 10.1016/s0140-6736(02)08651-8. 10.1016/S0140-6736(02)08651-8 12049861

[5] Allum WH, Stenning SP, Bancewicz J (2009). Long-term results of a randomized trial of surgery with or without preoperative chemotherapy in esophageal cancer. J Clin Oncol.

[6] Burmeister BH, Smithers BM, Gebski V (2005). Surgery alone versus chemoradiotherapy followed by surgery for resectable cancer of the oesophagus: a randomised controlled phase III trial. Lancet Oncol.

[7] van Hagen P, Hulshof MC, van Lanschot JJ (2012). Preoperative chemoradiotherapy for esophageal or junctional cancer. N Engl J Med.

[8] Shapiro J, van Lanschot JJB, Hulshof M (2015). Neoadjuvant chemoradiotherapy plus surgery versus surgery alone for oesophageal or junctional cancer (CROSS): long-term results of a randomised controlled trial. Lancet Oncol.

[9] Mariette C, Dahan L, Mornex F (2014). Surgery alone versus chemoradiotherapy followed by surgery for stage I and II esophageal cancer: final analysis of randomized controlled phase III trial FFCD 9901. J Clin Oncol.

[10] Lordick F, Mariette C, Haustermans K (2016). Oesophageal cancer: ESMO Clinical Practice Guidelines for diagnosis, treatment and follow-up. Ann Oncol.

[11] Ajani JA, D'Amico TA, Almhanna K (2015). Esophageal and esophagogastric junction cancers, version 1.2015. J Natl Compr Canc Netw.

[12] Gilbert S, Gresham GK, Jonker DJ (2012). Impact of patient selection, disease progression, and adverse events on esophageal cancer outcomes after trimodality therapy. Ann Thorac Surg.

[13] Courrech Staal EF, Aleman BM, Boot H (2010). Systematic review of the benefits and risks of neoadjuvant chemoradiation for oesophageal cancer. Br J Surg.

[14] Edge SB, Byrd DR, Compton CC, et al. American Joint Committee on Cancer (AJCC): Cancer staging manual. 7th ed. New York: Springer, 2010. 10.1245/s10434-010-0985-4 20180029

[15] Blom RL, Schreurs WM, Belgers HJ (2011). The value of post-neoadjuvant therapy PET-CT in the detection of interval metastases in esophageal carcinoma. Eur J Surg Oncol.

[16] Hulshoff JB, Smit JK, van der Jagt EJ (2014). Evaluation of progression prior to surgery after neoadjuvant chemoradiotherapy with computed tomography in esophageal cancer patients. Am J Surg.

[17] Anderegg MC, de Groof EJ, Gisbertz SS (2015). 18F-FDG PET-CT after neoadjuvant chemoradiotherapy in esophageal cancer patients to optimize surgical decision making. PLoS One.

[18] Seppenwoolde Y, Lebesque JV, de Jaeger K (2003). Comparing different NTCP models that predict the incidence of radiation pneumonitis. Normal tissue complication probability. Int J Radiat Oncol Biol Phys.

[19] Yorke ED, Jackson A, Rosenzweig KE (2005). Correlation of dosimetric factors and radiation pneumonitis for non-small-cell lung cancer patients in a recently completed dose escalation study. Int J Radiat Oncol Biol Phys.

[20] Hatakenaka M, Yonezawa M, Nonoshita T (2012). Acute cardiac impairment associated with concurrent chemoradiotherapy for esophageal cancer: magnetic resonance evaluation. Int J Radiat Oncol Biol Phys.

[21] Lund M, Alexandersson von Dobeln G, Nilsson M (2015). Effects on heart function of neoadjuvant chemotherapy and chemoradiotherapy in patients with cancer in the esophagus or gastroesophageal junction - a prospective cohort pilot study within a randomized clinical trial. Radiat Oncol.

[22] Beukema JC, van Luijk P, Widder J (2015). Is cardiac toxicity a relevant issue in the radiation treatment of esophageal cancer?. Radiother Oncol.

[23] He L, Chapple A, Liao Z (2016). Bayesian regression analyses of radiation modality effects on pericardial and pleural effusion and survival in esophageal cancer. Radiother Oncol.

[24] Whibley J, Peters CJ, Halliday LJ (2018). Poor performance in incremental shuttle walk and cardiopulmonary exercise testing predicts poor overall survival for patients undergoing esophago-gastric resection. Eur J Surg Oncol.

[25] McNair AG, Brookes ST, Kinnersley P (2013). What surgeons should tell patients with oesophago-gastric cancer: a cross sectional study of information needs. Eur J Surg Oncol.

[26] Stephens MR, Lewis WG, Brewster AE (2006). Multidisciplinary team management is associated with improved outcomes after surgery for esophageal cancer. Dis Esophagus.

[27] Rijksinstituut voor ziekte- en invaliditeitsverzekering dgv, verzekeringscomite. Nota cgv 2018/408 betreffende de uitvoering van punt 4.1.3.2 “complexe kankerzorg” van het nationaal akkoord artsen-ziekenfondsen 2018-2019. *Bijlage 1: complexe chirurgie bij slokdarmtumoren, gastro-oesofagale junctietumoren en niet-oncologische aandoeningen van de slokdarm*. Brussel; 2018. https://www.asgb.be/sites/default/files/2018-12/RIZIV_CGV_2018_408_Complexe%20chirurgie.pdf.

